# A pilot randomized controlled trial investigating the effects of an anti-inflammatory dietary pattern on disease activity, symptoms and microbiota profile in adults with inflammatory bowel disease

**DOI:** 10.1038/s41430-024-01487-9

**Published:** 2024-08-10

**Authors:** Abigail Marsh, Veronique Chachay, Merrilyn Banks, Satomi Okano, Gunter Hartel, Graham Radford-Smith

**Affiliations:** 1 The School of Human Movement and Nutrition Sciences, The University of Queensland, St Lucia, QLD Australia; 2 Department of Nutrition and Dietetics, Royal Brisbane and Women’s Hospital, Herston, QLD Australia; 3 Statistics Unit, QIMR Berghofer Medical Research Institute, Herston, QLD Australia; 4 School of Public Health, The University of Queensland, Brisbane, QLD Australia; 5 School of Nursing, Queensland University of Technology, Brisbane, QLD Australia; 6 Gut Health Group, QIMR Berghofer Medical Research Institute, Herston, QLD Australia; 7 Department of Gastroenterology and Hepatology, Royal Brisbane and Women’s Hospital, Herston, QLD Australia; 8 Faculty of Medicine, University of Queensland, Brisbane, Australia

**Keywords:** Inflammatory bowel disease, Randomized controlled trials, Chronic inflammation

## Abstract

**Background/Objectives:**

There is a lack of certainty in dietary prescription for individuals with inflammatory bowel disease (IBD) due to heterogeneity in studies to date. The aim of this study was to investigate the efficacy on disease activity of a modified anti-inflammatory dietary pattern purposely designed to reduce intake of food additives (IBD-MAID), compared to standard care, in adults with IBD.

**Subject/Methods:**

Adults with IBD were randomised to IBD-MAID (meals provided) [*n* = 29] or general healthy eating (GHE) [*n* = 29] for 8 weeks. Disease activity, faecal calprotectin (FC), C-reactive protein (CRP), symptoms, and quality of life (S&QOL) were assessed using validated tools.

**Results:**

The IBD-MAID was well tolerated and adhered to (92% adherence). At week 8, there was no statistically significant difference in change from baseline in outcome measures between groups. However, baseline to week 8 analysis indicated: (1) statistically significant improvements in S (*p* = 0.001) & QOL (*p* = 0.004), FC (*p* = 0.007), and Crohn’s disease activity (*p* = 0.03) but not ulcerative colitis, in individuals following the IBD-MAID and (2) statistically significant improvement in QOL in individuals receiving GHE (*p* = 0.015). Correlation analysis on change from baseline to week 8 revealed a greater decrease in food additives intake was associated with statistically significant improvements in FC, S & QOL and alignment of anti-inflammatory dietary principles with improvements in QOL.

**Conclusion:**

The IBD-MAID was well tolerated. The most novel finding pertains to the correlation between reduced food additives intake and improvements in inflammatory markers, S&QOL. Further research is needed to explore the effects of food additives exposure on IBD course.

**Trial registration:**

12619001500145

## Introduction

Increasing evidence suggests that diet plays an important role in the pathogenesis of inflammatory bowel disease (IBD). The incidence of IBD worldwide has paralleled the adoption of an ultra-processed Western style dietary pattern [1, 2]. Moreover, previous research has shown that diet is the major lifestyle factor modified by individuals with IBD to manage their symptoms [3]. Yet, current dietary guidelines for individuals with IBD are non-specific. This is largely due to a lack of well-designed, adequately powered, randomised controlled trials [4].

The traditional Mediterranean diet (TMD), is well recognised for its preventive properties towards chronic disease through the modulation of inflammation and beneficial effects on gut microbiota (GM) diversity and abundance [5–7]. Conversely, an ultra-processed dietary pattern (UPD) has been shown to correlate with higher circulating inflammatory cytokines and markers of oxidative stress in adults with chronic disease [8, 9]. Characteristic of an UPD includes the regular consumption of food additives such as artificial sweeteners, emulsifiers, and carrageenan gum. A growing body of evidence from in vitro and animal models suggests that these commonly used food additives damage the mucous layer of the gut wall and perturb the microbial ecosystem [10]. However, the application of findings to individuals with IBD is not known.

Hence, the aim of this pilot intervention study was to determine the safety, efficacy, and feasibility of following for eight weeks a dietary prescription developed to reduce exposure to pro-inflammatory dietary components and food additives, on disease activity, inflammatory markers, symptom severity, and quality of life, compared to standard care, in adults with IBD. The dietary pattern was termed the IBD modified anti-inflammatory diet (IBD MAID), and designed to contain low amounts of artificial sweeteners, emulsifiers, carrageenan gum, and maltodextrin.

## Methods

This pilot randomised controlled trial was conducted in accordance with the Consolidated Standards of Reporting Trials (CONSORT) guidelines. The CONSORT checklist is provided in the supplementary materials.

### Study setting and design

**Fig. 1:**
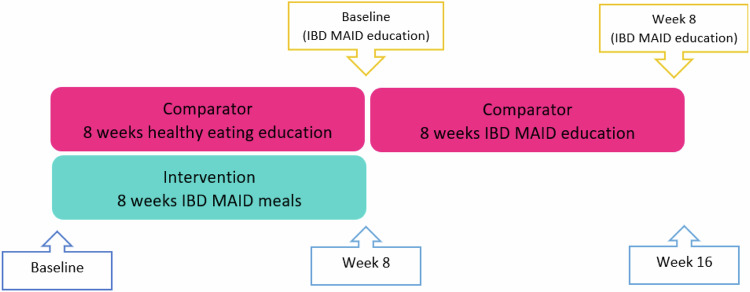
Key study timepoints - IBD MAID meals vs IBD MAID education.

This randomised trial spanned over 16 weeks. The first eight weeks compared the effectiveness of the IBD MAID delivered by meal provision and education of dietary principles (Intervention), with general healthy eating advice (Comparator group), on symptoms and inflammatory markers in adults with IBD. From weeks 8–16, participants in the Comparator group were assigned to the IBD MAID but delivered by education of principles and provision of key ingredients (extra virgin olive oil, unsalted nuts, and probiotic yoghurt) (Fig. 1).

Study participants were recruited from IBD outpatient clinics at a large tertiary hospital in Queensland, Australia. Participants were also referred to the study by local private gastroenterologists or were able to self-refer by emailing the primary investigator (PI). All participants were required to reside within a 20 km radius of the hospital for the purpose of meal delivery. The study was conducted between September 2019 and January 2022. This study followed the guidelines laid down in the Declaration of Helsinki and all procedures involving human subjects were approved by the Hospital and University of Queensland human ethics research committees (HREC/2019/QRBW/57618). The study was registered with the Australian New Zealand Clinical Trial Registry (ACTRN: 12619001500145). Written informed consent was obtained from all subjects prior to randomisation.

### Inclusion and exclusion criteria

Adult patients (18 to 60 years) with established UC or CD diagnosed by a gastroenterologist using current standard criteria, reporting a deterioration in symptoms at the time of their clinic appointment, with a faecal calprotectin (FC) of over 50 μg/mg were eligible to participate. Participants were excluded if they: could not speak or understand English, had a lack of capacity to provide informed consent, were current smokers or ceased smoking within 12 months prior to providing consent, were pregnant or lactating, had specific food allergies or intolerances which could hinder ability to follow diet prescription, had an ostomy, or had current stricturing and/or penetrating CD likely to need surgery within six months. Participants were also required to be on stable medical therapy at time of consent. Exclusions were: thiopurines, methotrexate or Janus kinase inhibitors started or with a change in dose less than eight weeks prior to enrolment, anti-TNF drugs started or a change in dose less than 12 weeks prior to enrolment, vedolizumab or other non-anti-TNF biological therapy started or change in dose less than 16 weeks prior to enrolment and a change in corticosteroid dose within four weeks, or a daily dose of ≥20 mg per day of prednisone or equivalent at time of screening.

### Randomisation and intervention

Participants were randomised one to one to Intervention or Comparator group stratified by disease subtype (UC or CD) and gender.

Individuals randomised to the Intervention group received two meals (lunch and dinner) daily for eight weeks, education on the IBD MAID principles, and recipes for breakfast and snacks. Meals were prepared and delivered by the catering company, ‘Wandering Italian' from recipes developed by the study dietitian. Participants were advised to eat until they were full. Larger or more active participants were encouraged to consume additional food items from the list of approved snacks. This group is referred to as IBD MAID-meals throughout the report.

Individuals in the Comparator group watched a video recording on general healthy eating (GHE) and were provided with recipes and education material from the ‘Eat for Health,’ Australian government public health nutrition initiative [11]. After the eight-week intervention period, individuals receiving GHE were assigned to the IBD MAID diet delivered by education on the diet principles, with provision of recipes, extra virgin olive oil, almonds, and probiotic yoghurt for eight weeks. This group was used to assess the real-life feasibility of the dietary pattern and is referred to as IBD MAID-education throughout the report.

### Outcome measures

Primary and secondary outcomes were measured at baseline, eight, and sixteen weeks. The primary outcome was change in disease activity from baseline to week 8. The Simple Clinical Colitis Activity Index (SCCAI) [12] score was used to determine UC disease activity and the Crohn’s Disease Activity Index (CDAI) [13] was used to determine CD activity. Scores were analysed as a continuous variable (from 0 to 19 for SCCAI and from 0 to 600 for CDAI). Secondary outcomes included quality of life, measured by the short inflammatory bowel disease questionnaire (SIBDQ) and symptom severity, assessed by the patient reported outcome 2 (PRO2) questionnaire. Intestinal inflammation was measured using faecal calprotectin (log transformed) and general inflammation was measured using C-reactive protein (CRP). A CRP response was defined as a CRP < 5 mg/L at week 8 or a reduction of ≥50% from baseline.

### Diet adherence assessment

Dietary adherence was assessed every four weeks via 3-day food diaries compiled on the Xyris Software ‘Easy Diet Diary’ app or on paper as preferred, and from a daily diet checklist designed for the study. IBD MAID dietary compliance was further assessed with the 14 point Mediterranean diet adherence screener (MEDAS) score (using ≥9 as compliance cut off) [14] and a food additive score developed specifically for the study (with a score of <3 indicating compliance) [Supplementary material].

### Food additives exposure assessment

To develop a food additives intake scoring method, five food additive sub-groups were identified based on the literature relating to food additives and gut health. A rate of frequency of dietary exposure for specific food additives was determined. The five-food additives sub-groups were: non-nutritive sweeteners, nitrites/nitrates from processed or smoked meats, maltodextrin, P80/CMC/carrageenan gum, and 'other' emulsifiers. The frequency of intake was calculated from three-day food diaries by adding the number of instances of intake. A scoring system was developed based on the number of instances of intake over the three days for each of the five food additive sub-groups: 0 for none to one instance, 1 for two to four instances, and 2 for greater than four occurrences. The total food additive score ranged from zero (very low food additive consumption) to ten (very high food additive consumption) across the three days.

A food additive score of less than three was determined as compliance to the dietary prescription.

### Statistical methods and power

All analyses were performed on a modified intention-to-treat basis using Stata 18 (StataCorp. 2023. Stata Statistical Software: Release 18. College Station, TX: StataCorp LLC.). Plots were generated using R software (v4.1.0).

Participants who withdrew from the study before week 8 (*n* = 3 in the Comparator and *n* = 5 in the intervention group) were excluded from the final analysis due to missing data. Per-protocol analysis was also performed by excluding participants who either changed medication during intervention or did not comply with IBD MAID prescription (MEDAS score <9 or diet additive score ≥3 at the end of study).

Patient characteristics and outcomes were presented using descriptive statistics; normally distributed data by mean and standard deviation (SD) and skewed distributions by median and interquartile range (IQR). Binary and categorical variables were presented using counts and percentages.

An independent sample t-test was used as the primary analysis to compare change score in the primary and secondary outcome measures (week 8—baseline) between the Comparator and Intervention groups. Analysis of covariance (ANCOVA) using a baseline score as a covariate was also used to account for potential baseline imbalance. Estimated mean difference in the change score between groups with 95% confidence intervals (CIs) was reported. A paired *t*-test was used to compare scores at baseline and at week 8 in the primary and secondary outcome measures within each group.

Changes in outcome measures from baseline to week eight in the Intervention group and from baseline to week 16 in the Comparator group were compared using an independent sample *t*-test and ANCOVA using baseline score as a covariate. Spearman’s correlation analysis was also performed to examine the relationship between change in diet adherence (MEDAS score and food additives score) and change in outcome measures.

Based on the literature investigating how to best estimate sample size when the effect size for an endpoint in the population of interest is unknown, we aimed to recruit 50 participants. Whitehead and colleagues (2016) [15] concluded that for a main trial designed with 80% power and two-sided 5% significance, with a moderate effect size (0.8), a pilot sample size of 25 per treatment arm is sufficient to provide estimates of the SD with sufficient precision for planning the main study [15]. Based on previous reported attrition rates in lifestyle interventions we estimated a drop-out rate of 25%. Therefore, the recruitment target was 67 participants.

### Stool microbiome analysis

Stool samples were collected at specified timepoints for 16s RNA analysis. Stool DNA (sDNA) was isolated using a bead-beating protocol prior to DNA extraction, as previously described by Zakrzewski and colleagues (2019) [16]. A control sample was carried out during the analysis to check for environmental and stool kit contamination. Quantification of total sDNA was performed using a Nanodrop spectrophotometer. sDNA was then stored at –80 °C until use. 16s rRNA sequencing and metagenomics sequencing was performed by the Australian Centre for Ecogenomics.

The GM was analysed for all IBD patients and by disease subtype (CD/UC). To analyse microbiome changes raw sequenced reads were processed and taxonomically assigned using the cut adapt and DADA plugins in QIIME2 [17]. The overall microbial composition was ordinated in a principal coordinates analysis plot to assess the beta diversity between the samples in the different groups, disease status (UC, CD), and time-point. Supervised (e.g. RDA discriminant analysis) and unsupervised (e.g. ADONIS permutation-based MANOVA) multivariate methods were applied to identify associations between the overall microbial diversity and intervention groups. Linear mixed-effects models were used to detect differences in taxon abundance between sample groups. *P*-values from multiple testing procedures were corrected to control for a specified false discovery rate or Bonferroni’s method.

## Results

Two hundred and eighty potential participants were screened, of whom 165 failed to meet the eligibility criteria, 43 did not complete the required screening and 13 elected not to participate in the study after meeting the eligibility criteria but before being randomised.

**Fig. 2:**
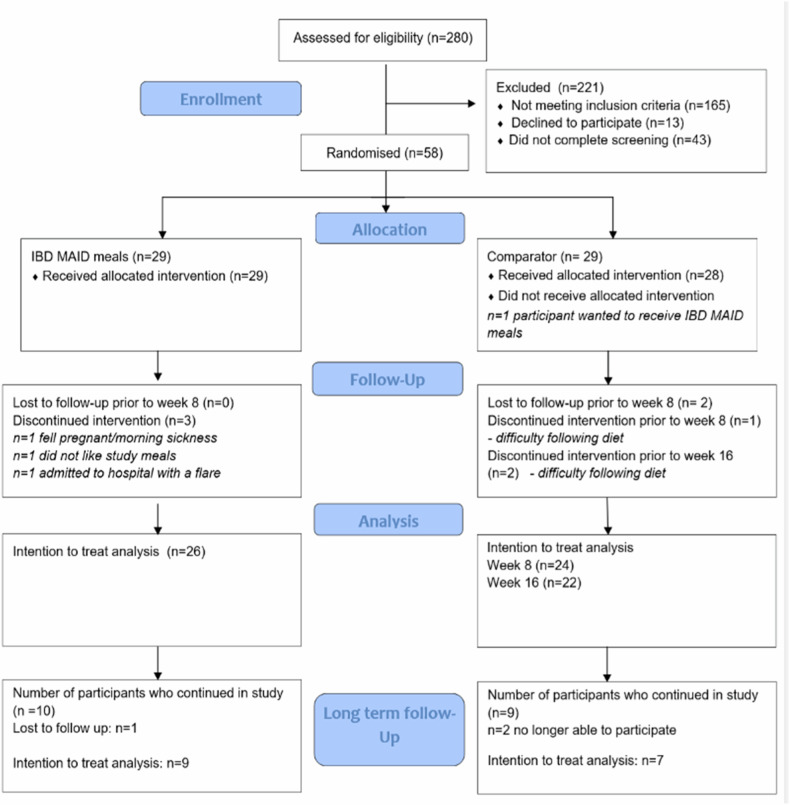
Recruitment, randomisation, and withdrawals. IBD MAID inflammatory bowel disease modified anti-inflammatory diet; *participants changed medication,** participants did not comply with diet prescription.

Twenty-nine participants were randomised to the Intervention group (IBD MAID meals) and 29 to the Comparator group (GHE). Three participants assigned to the IBD MAID meals group withdrew at weeks one and two. In the Comparator group, one participant withdrew post randomisation as they wanted to receive study meals, two participants were lost to follow-up at weeks two and four, and two participants elected not to continue due to difficulties with following diet prescription. At week 8, at commencement of the IBD MAID education prescription, one participant elected not to continue in the study and one participant withdrew at week three due to difficulty with following the education prescription (Fig. 2).

### Baseline descriptive characteristics

Fifty-three percent of participants were women, 82% from Caucasian descent, 10% from multi-racial origin, 6% from Asian ethnicity, and 4% identified as Indigenous Australian. The median body mass index was 25.5 (IQR 23.1–27.1) kg/m^2^, 65% of participants had never smoked and all participants reported having a secondary school education or higher. Covariates were generally well balanced between groups, however, more participants in the Comparator group were following a specific diet prescription at baseline (38% Comparator vs 20% Intervention), although this difference was consistent with random chance (n.s. *p* = 0.74).

### Baseline medical characteristics

Seventy-five percent of study participants were diagnosed with UC and 25% of participants were diagnosed with CD. Covariates were well balanced between groups. The median time since IBD diagnosis was 6.4 (IQR 2.4–14.7) years. All participants were prescribed at least one medication for IBD. Most participants with UC had pancolitis (53%) and were prescribed aminosalicylates (92%). Forty-six percent of participants had disease affecting the ileum and 31% had ileocolonic CD. Most participants with CD were prescribed immunomodulators (61%) and three participants (23%) had undergone resection surgery [Supplementary material].

Outcome measures at baseline were similar in the Comparator and Intervention groups. Participants in the Intervention group had a lower mean CDAI score (non-significant difference with Comparator) (Table 1).

**Table 1:** Baseline disease activity scores.

Parameters	Comparator (GHE)		Intervention (IBD MAID meals)	
	*n*		*n*	
SCCAI^a^	18	4.6 ± 2.9	19	4.8 ± 2.7
CDAI^a^	6	142.5 ± 62.1	7	98.6 ± 42.3
PRO2^a^	24	7.0 ± 5.2	25	7.7 ± 6.2
SIBDQ^a^	24	4.9 ± 1.0	26	4.9 ± 0.9
CRP ≥ 5 (mg/L)^b^	23	22% (5)	23	39% (9)
FC (μg/g)^c^	24	250.4 (166.6–376.3)	26	246.6 (172.7–352.3)

*CDAI* Crohn’s disease activity index, *FC* faecal calprotectin, *GHE* general healthy eating, *IBD MAID meals* inflammatory bowel disease modified anti-inflammatory diet meals, *PRO2* patient reported outcome 2 score, *SCCAI* simple clinical colitis activity index, *SIBDQ* short inflammatory bowel disease questionnaire.

^a^Reported as mean ± SD.

^b^Reported as % (*n*).

^c^Reported as geometric mean (95% CI) a.

Baseline dietary intake was similar in both the Comparator and Intervention group. The mean food additives score in the Comparator and Intervention group was 3.4 ± 1.4 vs 3.3 ± 1.6, respectively. However, participants in the Intervention group consumed significantly less fluid (1640 ml ± 949 ml) compared to the Comparator group (2534 ml ± 1119 ml) and had a higher mean MEDAS score (7.14 ± 1.5 vs 6.2 ± 1.6) at baseline.

In regard to the GM analysis, a total of 2793 Amplicon Sequence Variants (ASVs) were detected; 98 ASVs were removed as not present in more than 0.1% abundance in any sample at baseline, leaving 2695 ASVs for analysis. At the genus level the most abundant taxa at baseline were *Bacteroides* (belonging to phylum Bacteroidota), followed by *Faecalibacterium* and *Blautia* (phylum Firmicutes). Multivariate analyses showed no significant differences in the composition or relative abundances of organisms of different species in samples from treatment groups (Intervention vs Comparator group, Manova, *P* = 0.78) at baseline. However, there was a significant difference in the microbiota profile of participants with UC and CD at baseline (Manova, *P* < 0.02).

### Primary outcome measure

The mean change in disease activity score (SCCAI) from baseline to week eight in participants with UC was –0.3 ± 3.3 (*n* = 18, *p* = 0.72) in Comparator group and –0.7 ± 2.5 (*n* = 19, *p* = 0.21) in Intervention group. The mean difference in change between group (Intervention—Comparator) was –0.5 (95% CI: –2.4, 1.5, *p* = 0.63). In participants with CD, disease activity score (CDAI) was reduced from baseline to week eight in both groups (change in CDAI, –22.6 ± 46.3, *n* = 5, *p* = 0.34 in Comparator, –46.0 ± 43.1, *n* = 6, *p* = 0.030 in Intervention) (Table 2).

**Table 2:** Comparison of change in activity score from baseline to week 8 between intervention groups.

	Comparator (GHE)		Intervention (IBD MAID meals)		Mean difference in change (95% CI)	*P-*value
	*n*	Mean (SD)	*n*	Mean (SD)		
Change in SCCAI (Week 0–8) Range: 0–19	18	–0.3 (3.3)	19	–0.7 (2.5)	–0.5 (–2.4, 1.5)	0.63
Change in CDAI (Week 0–8) Range: 0–600	5	–22.6 (46.3)	7	–46.0 (43.1)	–23.4 (–81.3, 34.5)	0.39

Data presented as means and standard deviation, student *t*-test performed between groups at week 8.

*CDAI* Crohn’s disease activity index, *SCCAI* simple clinical colitis activity index.

**Fig. 3:**
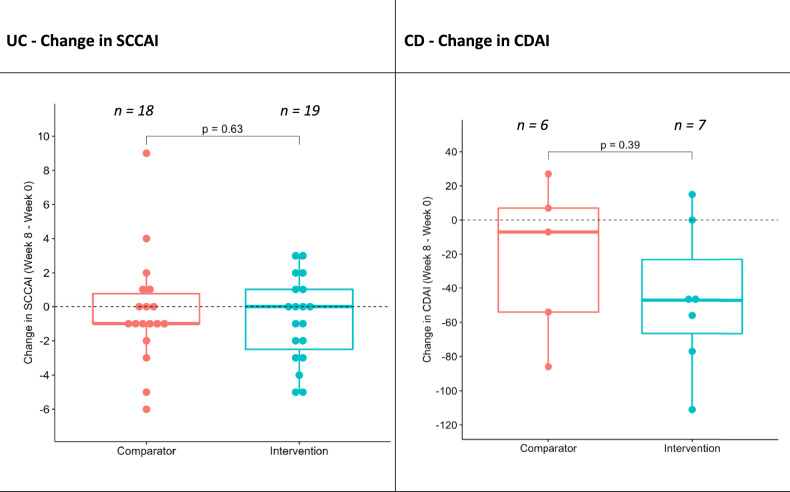
Change in disease activity scores from baseline to week 8. Each dot represents a participants change in disease activity across the study period. A student *t*-test was performed to compare change in both groups at week 8; CDAI Crohn’s disease activity index, SCCAI simple clinical colitis activity index.

Although the intervention group had a greater reduction on average, the difference between groups was not statistically significant (mean difference in change—23.4 (95% CI: –81.3, 34.5, *p* = 0.39)) (Fig. 3).

### Secondary outcome measures

#### Between groups analysis

**Fig. 4:**
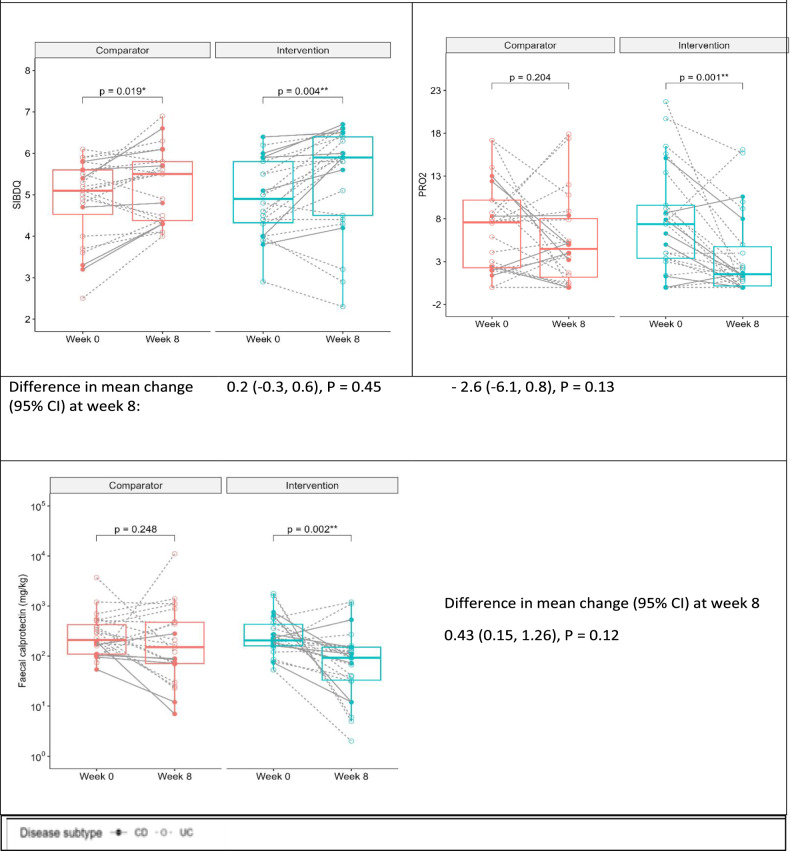
Comparison of change in secondary outcome measures from baseline to week 8. Each dot represents a participants change in disease activity across the study period. A student *t*-test was performed to compare change in both groups at week 8; CD Crohn’s disease, PRO2 patient reported outcomes 2 questionnaire, SIBDQ short inflammatory bowel disease questionnaire, UC ulcerative colitis.

At week eight, there were no statistically significant differences in change in any outcome measure between the groups (Fig. 4).

#### Within group analysis

In individuals in the Intervention group, from baseline to week eight, there was a statistically significant improvement in patient reported symptoms (change in PRO2 score, –4.3 ± 5.8, *P* = 0.001), quality of life (change in SIBDQ score, 0.5 ± 0.8, *P* = 0.004) and FC (geometric mean fold change 0.28,95% CI: 0.13, 0.59, *P* = 0.002).

In individuals in the Comparator group, there were statistically non-significant improvements in patient reported symptoms (–1.6 ± 6.2, *P* = 0.20) and FC (geometric mean fold change 0.64, 95% CI: 0.29,1.4, *P* = 0.25). There was a significant improvement in quality of life (0.3 ± 0.7, *P* = 0.019).

In the Intervention group, the proportion of participants achieving a CRP response from baseline to week eight was 73% and 65% in the Comparator group (OR 1.4, 95% CI: 0.4, 5.3) (Table 3).

**Table 3:** Comparison of change in secondary outcome measures from baseline to week 8.

Outcome measure	Comparator (GHE) (Week 0 to 8)		Intervention (IBD MAID meals) (Week 0 to 8)		Change between groups	
	*n*	Mean (SD)	*n*	Mean (SD)	Mean (95% CI)	*P*-value
Change in PRO2 score	24	–1.6 (6.2)	25	–4.3 (5.8)**	–2.6 (–6.1,0.8)	0.13
Change in SIBDQ score	24	0.3 (0.7)*	25	0.5 (0.8)**	0.2 (–0.3,0.6)	0.45
Change in FC (μg/g)†	23	0.64 (0.29–1.40)	25	0.28 (0.13–0.59)**	0.43 (0.15,1.26)	0.12
CRP response achieved, %(*n*)§	20	65% (13)	22	73% (16)	1.4 (0.4,5.3)	0.59

Data presented as means and standard deviations when normally distributed, counts and percentages when appropriate or †log-transformed data with geometric mean and 95% confidence intervals. Comparison of continuous variables from baseline to week eight in Comparator and Intervention group was performed by paired sample *t*-tests for normally distributed data or Wilcoxon signed-rank test for data not normally distributed and (§) Chi-squared test for categorical variables; significance set at set at *P < 0.05, **P < 0.01. Comparison of delta between groups (Comparator vs Intervention) was performed using an independent sample *t*-test; significance set at *p* < 0.05.

*CRP* C-reactive protein, *FC* faecal calprotectin, *PRO2* patient reported outcome 2, *SIBDQ* short inflammatory bowel disease questionnaire.

As an exploratory analysis, Spearman correlation analysis was performed to examine the relationship between change in diet adherence (MEDAS score and food additives score) and change in outcome measures from baseline to week 8 in all participants (Table 4). Change in MEDAS score from baseline to week eight was negatively correlated with fold change in FC (*r*_s_ = –0.334, *P* = 0.020). That is, a greater increase in MEDAS score from baseline to week eight was associated with a greater reduction (or smaller increase) in FC. The opposite was true for food additives score, which was positively correlated with fold change in FC, SIBDQ, and PRO2 from baseline to week eight. That is, a greater decrease in food additive scores was associated with a greater reduction in FC (*r*_s _= 0.35, *P* = 0.02) and PRO2 score (*r*_s_ = 0.33, *P* = 0.03) and a greater increase in SIBDQ score (*r*_s_ = –0.37, *P* = 0.01). This was also confirmed when reporting on UC participants alone (CD participants were not analysed separately due to small sample size). After correcting for multiple comparisons, a greater decrease in food additives score was associated with a greater increase in SIBDQ score (*r*_s_ = – 0.69, *P* = 0.03) and a greater reduction in FC (*r*_s _= 0.37, *P* = 0.05) and PRO2 score (*r*_s_ = 0.41, *P* = 0.04). There was no significant correlation between improvement in MEDAS score and outcome measures when analysing UC participants alone.

**Table 4:** Spearman correlation analysis performed to examine the relationship between change in diet adherence (MEDAS score and food additive score) and change in outcome measures from baseline to week 8.

Diet score	Outcome	*n*	Correlation coefficient (95% CI)	*p*.adj.BH
MEDAS	PRO2	49	–0.16 (0.43, 0.12)	0.310
MEDAS	SIBDQ	49	–0.09 (0.20,0.36)	0.553
MEDAS	FC	48	–0.33 (-0.56,–0.06)	0.039
Food additive	PRO2	47	–0.33 (0.04,0.56)	0.039
Food additive	SIBDQ	47	–0.37 (0.59,–0.09)	0.039
Food additive	FC	46	–0.35 (0.07,0.58)	0.039

Spearmen correlation analysis; data presented as correlation coefficient with 95% CI and adjusted *p*-values using the Benjamini & Hochberg method.

*CDAI* Crohn’s disease activity index, *FC* faecal calprotectin, *MEDAS* Mediterranean diet adherence screener, *PRO2* patient reported outcome 2 score, *SIBDQ* short inflammatory bowel disease questionnaire.

### Comparison of results in IBD MAID meals vs IBD MAID education

A comparison of outcome measures at week eight for individuals who received IBD MAID meals (Intervention group) with outcome measures at week 16 for individuals receiving eight weeks of GHE followed by eight weeks of IBD MAID education found no significant differences in any of the outcome measures between the two groups. Additionally, there was no significant difference in change in outcome measures between individuals receiving eight weeks of IBD MAID meals compared to individuals following eight weeks of IBD MAID education receiving key diet ingredients. [Supplementary material].

### Dietary adherence

Dietary adherence was determined by calculating the MEDAS score, and a food additives score developed for this study as described earlier.

The MEDAS score increased statistically significantly from baseline to week eight in the Intervention group (7.1 ± 1.5 to 10.5 ± 1.2, *P* < 0.001) and all but two participants (92%) met criteria for adherence (MEDAS ≥ 9). In the Comparator group, after 8 weeks of GHE education, the MEDAS score remained unchanged (6.2 ± 1.6 to 6.4 ± 1.7, *P* = 0.55). At week 16, after 8 weeks of IBD MAID education, the MEDAS score significantly increased (9.4 ± 1.7, *P* < 0.001) and all but seven participants (70%) met criteria for adherence to Mediterranean diet principles.

In the Intervention group, the food additives score decreased significantly from baseline to week eight (3.4 ± 1.4 to 0.2 ± 0.5, *P* < 0.001) and all participants met criteria for adherence to low food additives intake (food additive score < 3). Therefore, at week 8, 92% participants met overall dietary prescription adherence.

In the Comparator group, after 8 weeks of GHE education, the food additives score remained stable (3.3 ± 1.6 to 3.2 ± 1.5, *P* = 0.84). At week 16, after 8 weeks of IBD MAID education, the mean food additives score significantly decreased (2.6 ± 1.6, *P* = 0.036) and all but seven participants (70%) met the criteria for low food additives adherence. Overall, at week 16, 60% of participants in the IBD MAID education group met criteria for dietary prescription adherence.

### Gut microbiome

At week eight, there was no difference in the composition and/or relative abundances of organisms of different species in samples from both groups (MANOVA, *P* = 0.37). Furthermore, there was no significant change in diversity and richness from baseline to week eight (ANCOVA controlled for age and gender, *P* = 0.22) in either group. Age was the only variable that had a significant effect on diversity (*P* = 0.02). Between groups, analysis did not show any significant difference in change in microbial diversity or richness at the out level (mixed-effects linear regression, *P* = 0.25).

### Adverse events

The IBD MAID was well tolerated over the course of the study. During the intervention period, one participant at week three of receiving study meals was admitted to hospital for a disease flare, however, this was not deemed to be related to the intervention. This participant had reported meal tolerability up to the point of developing symptoms of a flare.

## Discussion

This pilot randomised controlled trial compared the safety, feasibility, and efficacy of following the IBD-MAID, compared to GHE, for eight weeks.

Study findings showed that the IBD-MAID was both well tolerated and well adhered to. After eight weeks of intervention, there were no statistically significant differences in the primary outcome measure of disease activity between the IBD-MAID and GHE groups. This is likely due to similarities in diet prescriptions with both diets improving overall diet quality by increasing fruit, vegetable, and wholegrain intake and decreasing intake of processed foods of participants. Indeed, from baseline to week 8, there was a statistically significant improvement in patient-reported symptoms, quality of life, systemic inflammatory markers, and CD activity score in the IBD-MAID group compared to baseline measures. In individuals following GHE there was also a statistically significant improvement in quality of life compared to baseline. Overall, both dietary interventions improved outcome measures from baseline to week 8. However, the greater improvement in outcome measures in individuals following the IBD MAID, albeit not statistically significant, supports a treatment effect as opposed to study findings resulting from a regression to the mean.

The study findings are comparable with results from a recent randomised controlled trial by Lewis and colleagues (2021) [18] who compared the TMD with the specific carbohydrate diet (SCD) on outcome measures in individuals with CD (*n* = 191). The SCD eliminated all grains, added sugars (except for honey), all milk products (except for hard cheeses and fermented dairy), and most ultra-processed foods. After six weeks of dietary intervention there was no statistically significant difference between groups. Within group analysis revealed statistically significant improvements in quality of life (QOL), patient-reported symptoms, and disease activity, *P* < 0.02 for all outcomes, in both groups. The authors suggested that the benefits of the dietary prescription were likely due to elimination of food additives as both diets were prepared from fresh ingredients. However, food additives consumption was not specifically quantified in the study.

Despite significant improvements in symptoms, QOL, inflammatory markers, and CD activity, there was no significant improvement in UC activity during the eight-week intervention in our study. No previous study has explored the effects of an anti-inflammatory diet in individuals with UC at the time of writing. At baseline, the mean SCCAI of study participants was 4.7 ± 2.7 (out of possible 19), indicating mild disease. Nine participants were in remission, 18 participants had mild disease and 10 participants had moderate disease. The large proportion of participants already in remission or with mild disease at baseline means that capturing improvement over 8 weeks was ambitious, especially in a small cohort. In the study by Lewis and colleagues (2021) [18] participants were excluded if they had low symptom burden. Future research should focus on individuals with greater disease activity at baseline to evaluate the effects of the IBD MAID on disease activity.

Analysis of the GM did not show any significant changes in diversity, ASVs, or taxa over the study duration. Lewis and colleagues (2021) also observed no change in richness and Shannon’s diversity indices over their total 12-week intervention in adults with CD following either the SCD or TMD. However, the authors did note a slight change in beta diversity, characterised by increased relative abundance of *Bacteroides vulgatus* and *Proteobacteria enterobacteriaceae* and decreased relative abundance of Firmicutes during the intervention. In the current study, the lack of effect on the GM profile is likely due to the study not being powered to assess significant changes in GM composition and the greater heterogeneity of the study population (UC and CD participants included).

The most important study finding pertains to the relationship between food additives intake, alignment with anti-inflammatory diet principles, and change in outcome measures. The IBD-MAID prescription was designed to be low in specific food additives (non-nutritive sweeteners, emulsifiers, maltodextrin, carrageenan gum, and nitrates) previously shown in animal and mechanistic models to perturb the GM and adversely affect gut health [19]. We found a significant positive correlation between reduction of exposure to food additives and improvement in FC, QOL, and patient-reported symptoms, whilst there was no effect on GM diversity or abundance. This result is noteworthy because the adherence to anti-inflammatory dietary principles, as determined by the MEDAS score, was only statistically significantly correlated with improvement in patient-reported symptoms. To our knowledge, this is the first study in individuals with UC to show a significant relationship between a reduction in food additives intake and improvement in both inflammatory markers and symptoms. Despite the observed benefit, educating patients on reducing food additives consumption is challenging. Currently, food manufacturers are not required to list the concentrations of food additives used on nutrition information panels in Australia [10]. Moreover, if an emulsifier is present within a compound ingredient that constitutes less than 5% of the final product it does not have to be identified on the ingredient list, which further adds to the complexity of identifying accurate food additives consumption [20]. Blanket recommendations of avoidance of ultra-processed foods may be warranted; but the use of food additives in the food supply is ubiquitous, and applies to everyday food items not usually considered as ultra-processed such as breads, wraps, and yoghurts. Future research is needed on validated methods to quantify food additive intake and subsequently the effects of food additives on gut health.

The meal provision in IBD MAID facilitated a greater control on the reduction of food additives. Indeed, a reduction of 100% was observed in the IBD MAID meals vs 68% IBD MAID education. The meal provision also likely influenced the more robust improvement in MEDAS score (92% IBD MAID meals versus 70% IBD MAID education). This resulted in a greater improvement in outcome measures in individuals receiving 8 weeks of IBD MAID meals (baseline to week 8) compared to individuals in the Comparator group receiving IBD MAID education alone for 8 weeks (week 8–16). However, this difference was not statistically significant between the groups. Drop-out rates were similar in both intervention arms. This shows that it is feasible to follow the IBD MAID prescription without meal provision and can be recommended in clinical practice. However, it is challenging to eliminate all food additives in practice due to their extensive use in the food industry and challenges in identification as aforementioned.

The IBD MAID prescription is rich in fermentable, oligo-, di-, monosaccharides, and polyols (FODMAP) containing foods. Previous research has shown that ~30–40% of individuals with IBD suffer from concurrent functional gut symptoms [21, 22]. In individuals with functional gut symptoms, consumption of FODMAP rich foods can worsen symptoms [23]. Study findings showed that similar numbers of participants reported an increase in both symptoms (*n* = 5, 10%), FC (*n* = 5, 10%), and disease activity (*n* = 7, 14%) in both intervention groups (IBD-MAID and GHE). This suggests that an increase in symptoms was not necessarily associated with the increased FODMAP content but more likely due to the presence of gut inflammation as evidenced by the increase in FC and disease activity. Additionally, both the IBD MAID and GHE were high-fibre dietary prescriptions. Historically, a ‘low residue’ diet has been prescribed by clinicians for individuals with IBD to reduce stool output and help manage gastrointestinal symptoms [24]. Furthermore, patients are often not advised to re-introduce fibre in the diet, resulting in prolonged and unnecessary fibre restriction adversely effecting GM diversity and abundance [24]. In the current study, both diets were well tolerated with no participants withdrawing from the study due to gastrointestinal complaints specific to meals. This further adds to the evidence that there is no need for the routine restriction of dietary fibre in adults with active disease.

This study has several limitations. As this was a pilot study and most participants had mild disease, endoscopic examination was not considered clinically indicated and hence mucosal healing was not assessed. It is well recognised that the treatment of IBD should focus on achieving mucosal healing, defined as the absence of macroscopic mucosal inflammation and ulceration, as compared to changes in signs and symptoms alone [25, 26].

The study intervention period was eight weeks which raises the question whether that was enough time to see changes in outcome measures. Previous dietary intervention studies in individuals with IBD have ranged from ten days to one year, with most studies following-up participants for six weeks [27]. Statistically significant changes in outcome measures were observed in the Comparator group at weeks 8 and 16 weeks from baseline measures, demonstrating the positive effect of improving diet quality, but potentially diluting the effect of the Intervention diet.

This study was only powered for large effect sizes at it is a pilot study. Due to limitations with recruitment during the COVID-19 pandemic and time constraints, we were unable to meet our recruitment target of *n* = 67 participants. Future studies should look at detecting smaller effect sizes. Furthermore, after running the study we noticed that CD was largely underpowered for statistical analysis due to unexpected challenges recruiting participants with CD.

## Conclusion

In conclusion, the IBD MAID was well tolerated among adults with UC and CD. Participants were able to adhere well to IBD MAID diet principles (IBD MAID meals and IBD MAID education with provision of key diet ingredients). At week eight, the IBD MAID was not shown to be superior to GHE; there was no statistically significant difference in outcome measures between the two groups. However, within group analysis, between baseline and week eight measures, found that there was a significant improvement in inflammatory markers, patient-reported symptoms, QOL, and disease activity (CD patients only) in individuals receiving IBD MAID meals (Intervention group), whilst GHE (Comparator group) was only associated with a significant improvement in QOL. The most novel study finding pertains to the significant correlation between a reduction in food additives consumption and improvements in FC, QOL, and patient-reported symptoms. Overall, study findings support a low food additives Mediterranean style dietary prescription and healthy eating for both individuals with CD and UC. Further research is needed to better understand the effects of food additives on human health and in UC more specifically as well as develop robust methods to quantify food additive intake. Additionally, more applied research is needed to develop tools that assist individuals in identifying the presence of food additives, inform food manufacturing regulations that empower individuals to be able to select appropriate low food additives options.

## Supplementary information

Supplementary materials

## Data Availability

The datasets generated during and/or analysed during the current study are available from the corresponding author on reasonable request.
