# Supplementary material for: A pilot randomized controlled trial investigating the effects of an anti-inflammatory dietary pattern on disease activity, symptoms and microbiota profile in adults with inflammatory bowel disease
Source: Eur J Clin Nutr. 2024 Aug 10;78(12):1072–81. doi: 10.1038/s41430-024-01487-9 (PMC11611722; doi:10.1038/s41430-024-01487-9)
Supplement: Supplementary file 1 — Supplementary materials [file 41430_2024_1487_MOESM1_ESM.docx]

**SUPPLEMENTARY MATERIAL**

**a)**

**
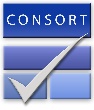
CONSORT 2010 checklist of information to include when reporting a randomised trial***

| **Section/Topic** | **Item No** | **Checklist item** | **Reported on page No** |
| --- | --- | --- | --- |
| **Title and abstract** | | | |
|  | 1a | Identification as a randomised trial in the title | 1 |
|  | 1b | Structured summary of trial design, methods, results, and conclusions (for specific guidance see CONSORT for abstracts) | 2 |
| **Introduction** | | | |
| Background and objectives | 2a | Scientific background and explanation of rationale | 4 |
|  | 2b | Specific objectives or hypotheses | 4 |
| **Methods** | | | |
| Trial design | 3a | Description of trial design (such as parallel, factorial) including allocation ratio | 5 |
|  | 3b | Important changes to methods after trial commencement (such as eligibility criteria), with reasons | 6 |
| Participants | 4a | Eligibility criteria for participants | 6 |
|  | 4b | Settings and locations where the data were collected | 5 |
| Interventions | 5 | The interventions for each group with sufficient details to allow replication, including how and when they were actually administered | 6, 7 |
| Outcomes | 6a | Completely defined pre-specified primary and secondary outcome measures, including how and when they were assessed | 7, 8 |
|  | 6b | Any changes to trial outcomes after the trial commenced, with reasons | N/A |
| Sample size | 7a | How sample size was determined | 9 |
|  | 7b | When applicable, explanation of any interim analyses and stopping guidelines | N/A |
| Randomisation: |  |  | 6 |
| Sequence generation | 8a | Method used to generate the random allocation sequence |  |
|  | 8b | Type of randomisation; details of any restriction (such as blocking and block size) | 6 |
| Allocation concealment mechanism | 9 | Mechanism used to implement the random allocation sequence (such as sequentially numbered containers), describing any steps taken to conceal the sequence until interventions were assigned | N/A |
| Implementation | 10 | Who generated the random allocation sequence, who enrolled participants, and who assigned participants to interventions | 6 |
| Blinding | 11a | If done, who was blinded after assignment to interventions (for example, participants, care providers, those assessing outcomes) and how | N/A |
|  | 11b | If relevant, description of the similarity of interventions | 6 |
| Statistical methods | 12a | Statistical methods used to compare groups for primary and secondary outcomes | 8 |
|  | 12b | Methods for additional analyses, such as subgroup analyses and adjusted analyses | 8 |
| **Results** | | | |
| Participant flow (a diagram is strongly recommended) | 13a | For each group, the numbers of participants who were randomly assigned, received intended treatment, and were analysed for the primary outcome | 10 |
|  | 13b | For each group, losses and exclusions after randomisation, together with reasons | 10, 11 |
| Recruitment | 14a | Dates defining the periods of recruitment and follow-up | N/A |
|  | 14b | Why the trial ended or was stopped | N/A |
| Baseline data | 15 | A table showing baseline demographic and clinical characteristics for each group | 11, 12 |
| Numbers analysed | 16 | For each group, number of participants (denominator) included in each analysis and whether the analysis was by original assigned groups | 13 |
| Outcomes and estimation | 17a | For each primary and secondary outcome, results for each group, and the estimated effect size and its precision (such as 95% confidence interval) | 13-15 |
|  | 17b | For binary outcomes, presentation of both absolute and relative effect sizes is recommended | yes |
| Ancillary analyses | 18 | Results of any other analyses performed, including subgroup analyses and adjusted analyses, distinguishing pre-specified from exploratory | 14 – 17 |
| Harms | 19 | All important harms or unintended effects in each group (for specific guidance see CONSORT for harms) | 18 |
| **Discussion** | | | |
| Limitations | 20 | Trial limitations, addressing sources of potential bias, imprecision, and, if relevant, multiplicity of analyses | 21-22 |
| Generalisability | 21 | Generalisability (external validity, applicability) of the trial findings | 22-23 |
| Interpretation | 22 | Interpretation consistent with results, balancing benefits and harms, and considering other relevant evidence | 22-23 |
| **Other information** | | |  |
| Registration | 23 | Registration number and name of trial registry | 2 |
| Protocol | 24 | Where the full trial protocol can be accessed, if available | N/A |
| Funding | 25 | Sources of funding and other support (such as supply of drugs), role of funders | 2-3 |

Citation: Schulz KF, Altman DG, Moher D, for the CONSORT Group. CONSORT 2010 Statement: updated guidelines for reporting parallel group randomised trials. BMC Medicine. 2010;8:18.
© 2010 Schulz et al. This is an Open Access article distributed under the terms of the Creative Commons Attribution License (<http://creativecommons.org/licenses/by/2.0>), which permits unrestricted use, distribution, and reproduction in any medium, provided the original work is properly cited.

*We strongly recommend reading this statement in conjunction with the CONSORT 2010 Explanation and Elaboration for important clarifications on all the items. If relevant, we also recommend reading CONSORT extensions for cluster randomised trials, non-inferiority and equivalence trials, non-pharmacological treatments, herbal interventions, and pragmatic trials. Additional extensions are forthcoming: for those and for up-to-date references relevant to this checklist, see [www.consort-statement.org](http://www.consort-statement.org).

***b) Development of food additive score***

Five food additive sub-groups were established considering the literature on food additives and gut health and frequency of exposure to specific food additives in the diet. The five-food additive sub-groups were: non-nutritive sweeteners, nitrites/nitrates, maltodextrin, P80/CMC/carrageenan gum, and other emulsifiers.

Intake of aforementioned food additives was calculated from three-day food diaries. A database of food additives found in all food items in the three-day food diaries was created to assist in the identification of food additives intake. This database expanded on work commenced by Trackman and colleagues (2020) who created a database of food additives in food items consumed by participants of the ‘Enigma study.’^20, 29^. The database created by Trackman and colleagues contained 145 food items with the additives as listed on the nutrition information panel. If food items consumed in participants’ food diaries were not listed in the database, they were added to this database. The final database consisted of 1524 food items. If participants did not report the brand of a particular food item, the brand was assumed to be a common Australian brand for that product. A list of assumptions was created for consistency.

*Supplementary Table 1: Food additive scoring table*
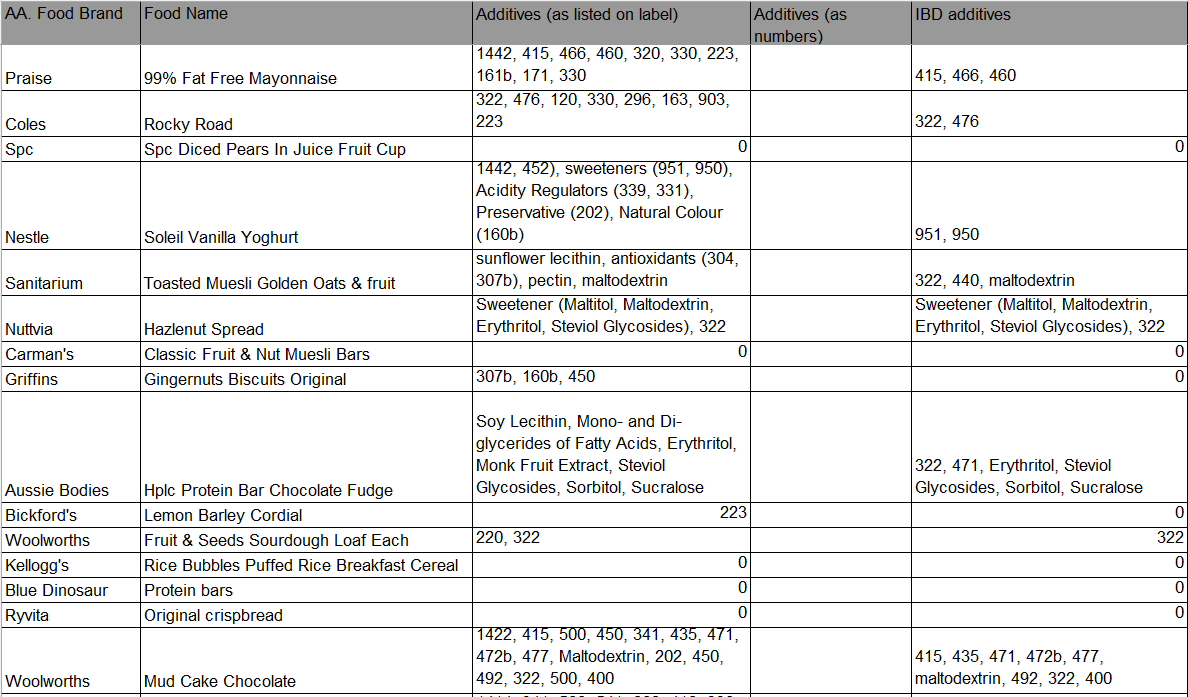
From the database, a scoring system was developed to categorise food additives intake. Participants who consumed additives from each subgroup nil or one time across the three-days were allocated a zero, two to four times were allocated one, and participants who consumed the food additives on more than five occurrences were allocated two. Therefore, the total food additive score ranged from zero to ten across the three days.

A food additive score of less than three was determined as dietary compliance to the study dietary prescription. This was determined from face to face validity testing at different scores e.g. 0, 1, 2, 3, 4).

***c) Medical characteristics of participants stratified by disease subtype***

*Supplementary Table 2: Medical characteristics of participants with ulcerative colitis at baseline*

|  | **Comparator (GHE)**  (n = 18) | **Intervention (IBD MAID meals)** (n=19) |
| --- | --- | --- |
| Time since UC diagnosis (years), median (IQR) | 6.5 (2.3 -14.7) | 6.8 (2.5-15.7) |
| Extent of disease |  |  |
| *Ulcerative proctitis (E1), n(%)* | 4 (22) | 5 (26) |
| *Distal UC (E2), n(%)* | 4 (22) | 5 (26) |
| *Pancolitis (E3), n(%)* | 10 (56) | 9 (47) |
| Resection surgery | 0 (0) | 0 (0) |
| History of extra-intestinal manifestation, n (%) | 6 (33) | 8 (42) |
| *Arthritis* | 5 (28) | 5 (26) |
| *Iritis* | 1 (6) | 2 (11) |
| *Skin/mouth lesions* | 3 (17) | 3 (16) |
| Current UC medications |  |  |
| *Oral corticosteroids* | 2 (11) | 3 (16) |
| *Corticosteroid suppositories* | 4 (23) | 1 (5) |
| *Aminosalicylates* | 16 (89) | 19 (100) |
| *Immunomodulators* | 4 (23) | 2 (11) |
| *Biologics* | 3 (17) | 4 (21) |
| *Laxatives* | 3 (17) | 2 (11) |

*Supplementary Table 3: Medical characteristics of participants with Crohn’s disease at baseline*

|  | **Comparator (GHE)** (n=6) | **Intervention (IBD MAID meals)** (n=7) |
| --- | --- | --- |
| Time since CD diagnosis (years), median (IQR) | 5.6 (1.8-13.4) | 6.8 (2.5-15.7) |
| Extent of disease n (%) |  |  |
| *Ileum (L1)* | 3 (50) | 3 (43) |
| *Colon (L2)* | 2 (33) | 1 (14) |
| *Ileocolon (L3))* | 1 (17) | 3 (43) |
| *Upper gastrointestinal (L4)* | 0 (0) | 0 (0) |
| CD behaviour n (%) |  |  |
| *Non-stricturing, non-penetrating (B1)* | 4 (67) | 6 (86) |
| *Stricturing (B2)* | 1 (17) | 1 (14) |
| *Penetrating (B3)* | 2 (33) | 0 (0) |
| *Perianal disease* | 1 (17) | 2 (29) |
| Resection surgery | 2 (33) | 1 (14) |
| History of extra-intestinal manifestation, n (%) | 2 (33) | 1 (14) |
| *Arthritis* | 1 (17) | 0 (0) |
| *Iritis* | 0 (0) | 0 (0) |
| *Skin/mouth lesions* | 1 (17) | 1 (14) |
| Current CD medications |  |  |
| *Oral corticosteroids* | 0 (0) | 0 (0) |
| *Corticosteroid suppositories)* | 0 (0) | 0 (0) |
| *Aminosalicylates* | 1 (17) | 2 (29) |
| *Immunomodulators* | 4 (67) | 4 (57) |
| *Biologics* | 1 (17) | 2 (29) |
| *Laxatives* | 1 (17) | 0 (0) |

***d) Comparison of results in IBD MAID meals vs IBD MAID education***

*Supplementary Table 4: Change in parameters from week 8 to week 16 in Comparator group compared to change from baseline to week 8 in Intervention group*

| **Parameters** | **N** | **Comparator** (Week 8 - 16) | **n** | **Intervention** (Week 0 - 8) | **Mean difference** (95% CI) | **P-value** |
| --- | --- | --- | --- | --- | --- | --- |
| SCCAI^¶^ | 16 | -0.7 ± 3.7 | 19 | -0.7 ± 2.5 | -0.05 (-2.2,2.1) | 0.96 |
| CDAI^¶^ | 5 | -16.0 ± 56.4 | 7 | -46.0 ± 43.1* | -30.0 (-93.7,33.7) | 0.32 |
| PRO2 score^¶^ | 22 | -1.8 ± 6.1 | 25 | -4.3 ± 5.8** | -2.5 (-6.0,1.0) | 0.16 |
| SIBDQ score^¶^ | 21 | 0.3 ± 0.7 | 25 | 0.5 ± 0.8** | 0.2 (-0.2,0.6) | 0.38 |
| FC† | 21 | 0.57(0.29,1.11) | 25 | 0.57(0.29,1.11) | 0.49 (0.18,1.34) | 0.16 |
| CRP response achieved§ | 16 | 88% (14) | 22 | 73% (16) | 0.4 (0.1,2.2) | 0.28 |

^¶^ reported as mean change (SD) and tested using *independent sample t-test, †* reported as geometric mean fold change (95% CI) and tested using *independent sample t-test on log-transformed fold change*, *§ reported as % (n) and tested using logistic regression and presented as Odds ratio (95%CI). A paired sample t-test was used to compare in SCCAI, CDAI, PROS score, SIBDQ score and FC between baseline and week 8 for Intervention group or between week 8 and 16 for Comparator group (*P <0.05, **P < 0.01) CDAI: Crohn’s disease activity index; FC: Faecal Calprotectin; PRO2: Patient Reported Outcome 2 Score; SCCAI: Simple Clinical Colitis Activity Index; SIBDQ: Short Inflammatory Bowel Disease Questionnaire*
